# Surgical outcomes and risk factors for surgical complications after en bloc resection following reconstruction with 3D-printed artificial vertebral body for thoracolumbar tumors

**DOI:** 10.1186/s12957-023-03271-8

**Published:** 2023-12-14

**Authors:** Jinxin Hu, Guohui Song, Hongmin Chen, Huaiyuan Xu, Anqi Wang, Xiangqin Wang, Bingbing Hou, Jinchang Lu, Qinglian Tang, Jin Wang, Xiaojun Zhu

**Affiliations:** 1 Department of Musculoskeletal Oncology, Sun Yat-Sen University Cancer Center, Guangzhou, 510060 People’s Republic of China; 2 State Key Laboratory of Oncology in South China, Guangdong Provincial Clinical Research Center for Cancer, Sun Yat-Sen University Cancer Center, Guangzhou, 510060 People’s Republic of China

**Keywords:** Complication, En bloc resection, Spinal tumor, Thoracolumbar spine, 3D-printed artificial vertebral body

## Abstract

**Background:**

The outcomes of patients with tumors of the thoracolumbar spine treated with en bloc resection (EBR) using three-dimensional (3D)-printed endoprostheses are underreported.

**Methods:**

We retrospectively evaluated patients with thoracolumbar tumors who underwent surgery at our institution. Logistic regression analysis was performed to identify the potential risk factors for surgical complications. Nomograms to predict complications were constructed and validated.

**Results:**

A total of 53 patients with spinal tumors underwent EBR at our hospital; of these, 2 were lost to follow-up, 45 underwent total en bloc spondylectomy, and 6 were treated with sagittal en bloc spondylectomy. The anterior reconstruction materials included a customized 3D-printed artificial vertebral body (AVB) in 10 cases and an off-the-shelf 3D-printed AVB in 41 cases, and prosthesis mismatch occurred in 2 patients reconstructed with the off-the-shelf 3D-printed AVB. The median follow-up period was 21 months (range, 7–57 months). Three patients experienced local recurrence, and 5 patients died at the final follow-up. A total of 50 perioperative complications were encountered in 29 patients, including 25 major and 25 minor complications. Instrumentation failure occurred in 1 patient, and no prosthesis subsidence was observed. Using a combined surgical approach was a dependent predictor of overall complications, while Karnofsky performance status score, lumbar spine lesion, and intraoperative blood loss ≥ 2000 mL were predictors of major complications. Nomograms for the overall and major complications were constructed using these factors, with C-indices of 0.850 and 0.891, respectively.

**Conclusions:**

EBR is essential for the management of thoracolumbar tumors; however, EBR has a steep learning curve and a high complication rate. A 3D-printed AVB is an effective and feasible reconstruction option for patients treated with EBR.

## Background

The management of spinal tumors requires multidisciplinary teams to work collaboratively, and surgery is the key to obtaining long-term local control. Over the last 30 years, surgical management strategies for spinal tumors have evolved from curettage and intralesional resection to en bloc resection (EBR) [1]. EBR is aimed at surgically extirpating the tumor as a whole, fully encased by a layer cuff of normal tissue, which is defined as the “margin,” and is of great importance for local tumor control [2]. Numerous studies have shown that EBR can significantly improve survival and local control compared with piecemeal resection for spinal malignancies, including primary tumors of the spine and isolated spinal metastases [3–6].

Nevertheless, EBR is a technically demanding procedure with a steep learning curve owing to the delicate nature of the surrounding anatomy. The complication rate of EBR remains relatively high, even when performed by experienced surgeons [7–9]. Boriani et al. [2] retrospectively investigated 220 patients treated with EBR and found a complication rate of 46.2%. Accordingly, the surgical complications of EBR should always be considered in the decision-making process. However, most reported clinical cases of EBR adopted conventional methods to perform anterior reconstruction, after which hardware problems and instrumentation failure occurred in up to 40% of patients [10, 11]. Furthermore, the outcomes of patients treated using 3D-printed endoprostheses have barely been reported.

In this retrospective study, we aimed to investigate the outcomes of patients with tumors of the thoracolumbar spine treated with EBR to identify surgical outcomes and predictors of surgical complications; all patients underwent reconstruction with a 3D-printed artificial vertebral body (AVB). To the best of our knowledge, this is the largest study of its kind to date.

## Materials and methods

### Inclusion criteria

Patients were selected according to the following criteria: (i) treatment conducted between 2017 and 2022, (ii) diagnosis of solitary spinal metastases or primary malignant and aggressive benign tumors of the spine verified by postoperative pathological examinations, (iii) treatment with EBR, and (iv) minimum of 6-month follow-up.

### Exclusion criteria

The exclusion criteria were as follows: (i) patients with multiple skip lesions (Tomita type 7) and (ii) tumors located in the cervical spine, cervical-thoracic junction, and sacrum.

## Methods

### General information

**Fig. 1:**
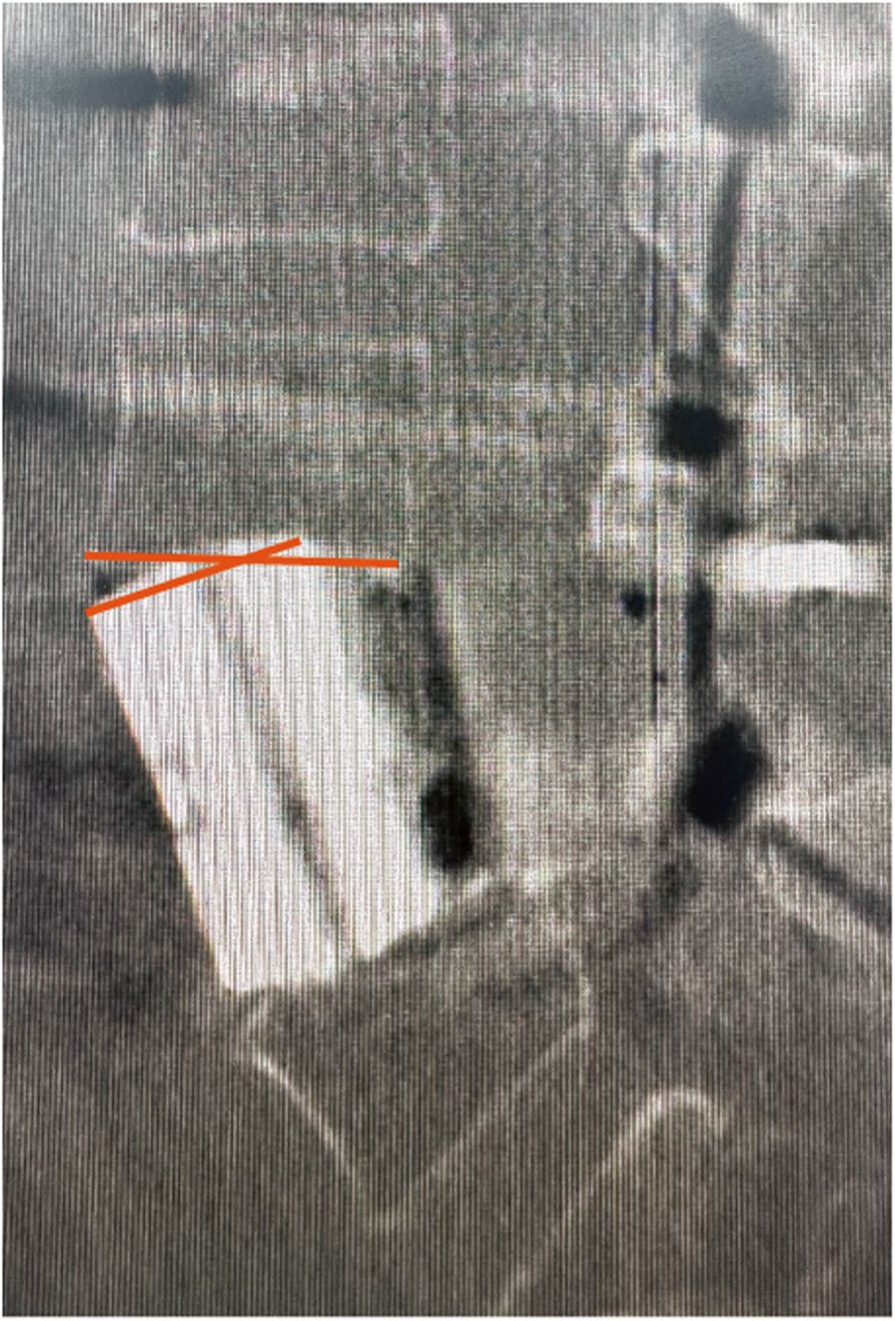
Mismatch of the prosthesis was defined as the angle between the endplate (or osteotomy plane) and the artificial vertebral body exceeding 10° on the immediate postoperative computed tomography scan

Hospital charts, pathology reports, operating room reports, and radiographic data were retrospectively reviewed. Study parameters included patient age, sex, comorbidities, radiological features, histology, adjuvant therapy, estimated blood loss, length of hospital stay, and surgical complications. Performance status was assessed using the Karnofsky Performance Status (KPS) score, and neurological function was evaluated using the American Spinal Injury Association Impairment Scale. Surgical margins were evaluated using pathology reports, and pain was evaluated using a visual analog scale (VAS). Mismatch of the AVB was defined as the angle between the endplate (or osteotomy plane) and the AVB exceeding 10° on immediate postoperative computed tomography (CT) scan (Fig. 1). Perioperative complications were classified either as major or minor, as described by McDonnel et al. [12]. Any complication that appeared to substantially alter the recovery process and increase the duration of hospitalization was described as a major complication, while other complications were regarded as minor. Complications were divided into intraoperative, early postoperative (occurring within the first 30 days after surgery), and late postoperative (occurring after 30 days following surgery).

### Treatment strategy

All patients underwent total en bloc spondylectomy (TES) or sagittal en bloc spondylectomy (SES), as proposed by Boriani et al. [2, 13]. For cases with suspected malignant tumors, the histological diagnosis was confirmed preoperatively by percutaneous needle biopsy or open minimally invasive biopsies. Individualized therapeutic strategies were tailored by a multidisciplinary team according to the patient’s health status and tumor size, location, and histology. Preoperative embolization was performed one day prior to the operation in selected patients to minimize intraoperative blood loss. Regarding the surgical plan, a personalized surgical approach and en bloc plan were developed according to the Weinstein-Boriani-Biagini surgical staging system. Relevant anatomical structures, such as nerve roots, were sacrificed to achieve disease-free margins, as needed. Internal fixation and/or fusion were performed to restore spinal continuity and stability after tumor resection, and anterior reconstruction was performed either using customized 3D-printed AVB or off-the-shelf 3D-printed AVB. For patients reconstructed with customized 3D-printed AVB, all prostheses were designed by our clinical team based on 1-mm thin-layer CT imaging and manufactured by AK MEDICAL Ltd., China. The prostheses were made of titanium alloy (Ti_6_Al_4_V) through electron beam melting technology (ARCAM Q10plus, Molndal, Sweden). In order to prevent positional alterations in spinal alignments and mismatch between the defect and the prostheses, 3 implants with a size difference of 3 mm were prepared for selected patients, especially when the tumor was located in the lower lumbar spine and upper thoracic spine. The prostheses were ethylene oxide-sterilized prior to implantation and the manufacturing process normally takes about 1 to 2 weeks.

### Follow-up

We routinely performed reconstructive CT and magnetic resonance imaging at 3, 6, 9, and 12 months postoperatively and every 6 months thereafter to monitor local control (LC). Patients also underwent radiographic examinations at any time when they developed signs or symptoms suggestive of tumor recurrence or metastasis. Positron emission tomography/CT was performed in some patients to explore distant metastases.

### Statistical analysis

Continuous variables are expressed as mean values ± standard deviation for those with a normal distribution or as the median (range) for those with non-normal distribution, and categorical variables are presented as counts (percentages). The normality of distribution was assessed using the Kolmogorov–Smirnov test. Time-to-event data was defined as the interval from the start date of surgery to that of the respective event (tumor recurrence and death) or the last follow-up. The Kaplan–Meier method was used to estimate local progression and overall survival (OS). Univariate logistic regression analysis was conducted to assess the significance of the potential predictors of interest. Multivariate logistic regression analysis was applied to determine the joint effect of potential factors (those with *P* < 0.05 on the univariate analysis). The nomogram function in the rms R package (v6.3–0) was used to plot the nomogram. Statistical significance was set at *P* < 0.05. All analyses were conducted using SPSS version 26 for Mac (IBM Corp., Armonk, New York, USA) and R software (v4.1.2).

## Results

### Patient and treatment characteristics

Between January 2017 and December 2022, fifty-three consecutive patients with tumors involving the thoracic or lumbar spine underwent EBR at our orthopedic department. The definitive diagnosis was verified by postoperative histopathological examination in all patients, and 2 patients were lost to follow-up. Finally, 51 patients were included in this study; the mean age of these patients at admission was 41.9 ± 16.0 years, and 31 (60.8%) were male (Table 1). There were 33 primary tumors (64.7%) and 18 metastatic tumors (35.3%). The most common histological diagnosis of primary tumors was giant cell tumor of the bone (*n* = 10, 19.6%), followed by osteosarcoma (*n* = 4, 7.8%), and aggressive vertebral hemangioma (*n* = 4, 7.8%). Patients with metastatic tumors most commonly presented with liver (*n* = 4, 7.8%) and thyroid cancers (*n* = 3, 5.9%) (Table 2).

**Table 1:** Summary of baseline and surgical characteristics

Variables	All patients (*n* = 51)
Gender	
Male	31 (60.8%)
Female	20 (39.2%)
Age *(year)*	
Mean ± stand. dev	41.9 ± 16.0
BMI *(kg/m*^*2*^*)*	
Mean ± stand. dev	22.6 ± 3.9
KPS score	
≥ 80	37 (72.5%)
50–70	12 (23.5%)
0–40	2 (3.9%)
Prior radiation treatment	4 (7.8%)
Prior systemic therapy	20 (39.2%)
Prior operation	7 (13.7%)
ASIA score	
A–C	5 (9.8%)
D	13 (25.5%)
E	33 (64.7%)
Location	
Thoracic	35 (68.6%)
Lumbar	16 (31.4%)
Operation segments	
Single	38 (74.5%)
Multiple	13 (25.5%)
Reconstruction	
Customized 3D-printed AVB	10 (19.6%)
Off-the-shelf 3D-printed AVB	41 (80.4%)
Surgical type	
TES	45 (88.2%)
SES	6 (11.8%)
Approach	
Single	35 (68.6%)
Combined	16 (31.4%)
Surgical time *(minutes)*	
Median (range)	387 (185–910)
Intraoperative blood loss *(mL)*	
Median (range)	1800 (500–7700)
Length of hospital stay *(days)*	
Median (range)	19 (7–96)

**Table 2:** Tumor histology

	Number (*n* = 51)
Primary	33 (64.7%)
Osteosarcoma	4 (7.8%)
Myofibrosarcoma	1 (2.0%)
Chondrosarcoma	2 (3.9%)
Plasmacytoma	2 (3.9%)
Synovial sarcoma	1 (2.0%)
Alveolar soft part sarcoma	1 (2.0%)
Undifferentiated pleomorphic sarcoma	1 (2.0%)
Schwannomas	1 (2.0%)
Giant cell tumor	10 (19.6%)
Aggressive hemangioma	4 (7.8%)
Solitary fibrous tumor	3 (5.9%)
Other	3 (5.9%)
Metastasis	18 (35.3%)
Breast	2 (3.9%)
Lung	2 (3.9%)
Liver	4 (7.8%)
Thyroid	3 (5.9%)
Cervix	1 (2.0%)
Other	6 (11.8%)

With regard to the tumor location, the thoracic spine was most commonly involved (*n* = 35, 68.6%), followed by the lumbar spine (*n* = 16, 31.4%). The lesions were found to erode 1 bony segment in 38 (74.5%) patients, 2 segments in 6 (11.8%) patients, 3 segments in 6 (11.8%) patients, and 5 segments in 1 (2.0%) patient. Of these patients, 18 (35.3%) presented with pathological fractures, and 7 (13.7%) showed symptoms of myelopathy. At presentation, the tumors of 7 (13.7%) patients had a preceding surgical therapy in another hospital, and 4 (7.8%) lesions had previously been irradiated; the median preoperative KPS score was 80 (range, 30–100).

A total of 37 (72.5%) patients underwent preoperative percutaneous needle biopsy or open minimally invasive biopsy, and 20 (39.2%) patients had received prior systemic therapy. TES was performed in 45 (88.2%) patients and SES in 6 (11.8%) patients. Preoperative embolization was performed in 6 (11.8%) patients treated with TES. The anterior reconstruction materials included customized 3D-printed AVB in 10 patients (19.6%) and off-the-shelf 3D-printed AVB in 41 patients (80.4%). Two patients with lumbar spine tumors had a prosthesis mismatch after the operation, all of which were reconstructed with the off-the-shelf 3D-printed AVB. Regarding the surgical approach, a one-stage single posterior approach operation was performed in 35 (68.6%) patients and a one-stage combined approach in 16 (31.4%). The median operation time was 387 (range, 185–910) min, and the median estimated blood loss and intraoperative red blood cell transfusion were 1800 (range, 500–7500) mL and 12 (range, 4.0–46.5) units, respectively. Of the 51 patients included, 11 (21.6%) received postoperative radiotherapy, and 24 (47.1%) received adjuvant systemic therapy. The median duration of hospital stay was 19 (range, 7–96) days.

### Complications

A total of 50 perioperative complications, including 25 major and 25 minor, were encountered in 29 (56.9%) patients. Five intraoperative complications were observed in 5 (9.8%) patients and 45 postoperative complications in 28 (54.9%) patients. Sixteen (31.4%) patients had a single complication, 8 (15.7%) had 2 complications, 4 (7.8%) had 3 complications, and 1 (2.0%) had 6 complications. With regard to intraoperative complications, 3 major and 2 minor complications were documented; the most commonly encountered intraoperative complication was massive bleeding in 2 (3.9%) patients, followed by injury to the vena cava, lung injury, and dural tear in 1 (2.0%) patient each. In the early postoperative period, the most frequent complications were pleural effusion, occurring in 10 (19.6%) patients, and deep wound infection, occurring in 9 (17.6%) patients. Five patients (9.8%) experienced neurological deterioration after surgery and other complications including urine leakage, chyle leakage, pneumothorax, pneumonia, hematoma, superficial wound infection, subdural hemorrhage, and deep venous thrombosis. Additionally, one patient experienced back pain due to a deep infection combined with screw backout during the follow-up period. The patient underwent a revision surgery and recovered well. Thirteen (25.5%) patients required at least one reoperation during the follow-up period, and the most common reasons for reoperation were wound-related complications, occurring in 9 (17.6%) cases, followed by massive hematoma in 4 (7.8%) cases. One patient underwent craniotomy for intracranial subdural hematoma evacuation. Fortunately, the patient recovered without sequelae and was discharged uneventfully. No intra- or perioperative mortality was observed (Table 3).

**Table 3:** Overall surgical complications

Complications	Number of cases (*n* = 50)
Intraoperative	5
Major	
Massive bleeding	2
Vena cava injury	1
Minor	
Dural tear	1
Lung injury	1
Early	44
Major	
Deep wound infection requiring surgical debridement	9
Neurological deterioration	5
Urine leakage	2
Displacement of prosthesis	1
Intracranial subdural hematoma	1
Hematoma	2
Pneumonia	1
Minor	
Pleural effusion	10
Cerebral-spinal leakage	4
Chyle leakage	3
Pneumothorax	1
Deep venous thrombosis	1
Tracheal obstruction	1
Pneumonia	1
Superficial wound infection	2
Late	1
Major	
Hardware failure requiring reoperation	1

### Risk factors of complications

**Fig. 2:**
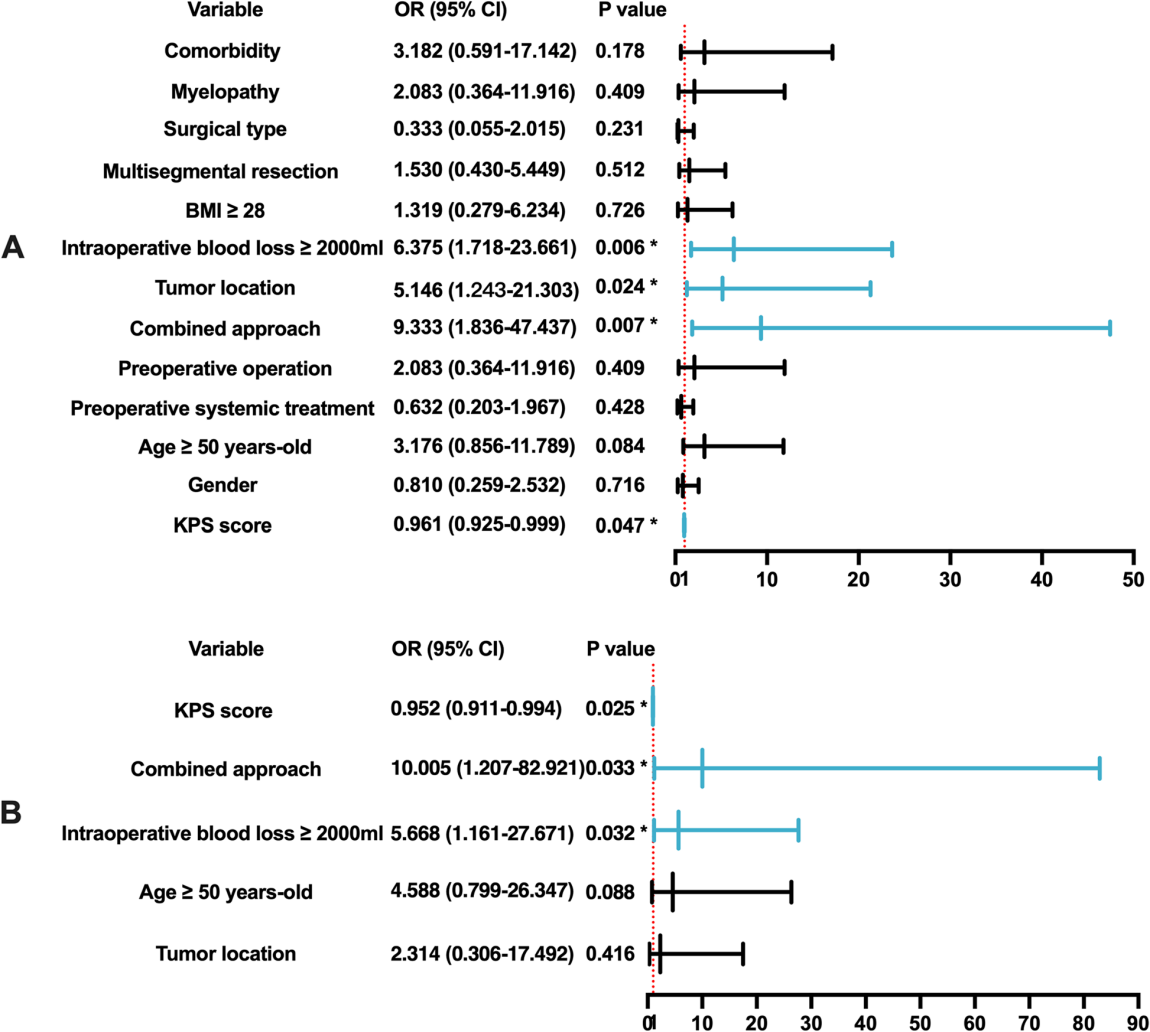
Predictors of overall complications by univariate and multivariate analysis. **A** Forest plot of the results of the univariate analysis. **B** Forest plot of the results of the multivariate analysis. Abbreviations: OR, odds ratio; CI, confidence interval; BMI, body mass index; KPS, Karnofsky performance status

Univariate analyses revealed combined surgical approach (*P* = 0.007), low KPS score (*P* = 0.047), intraoperative blood loss ≥ 2000 mL (*P* = 0.006), and lumbar spine lesion (*P* = 0.024) as significant risk factors for overall complication occurrence. In the multivariate logistic regression analysis, the KPS score (*P* = 0.025), combined surgical approach (*P* = 0.033), and intraoperative bleeding (*P* = 0.032) were associated with overall complications (Fig. 2).

**Fig. 3:**
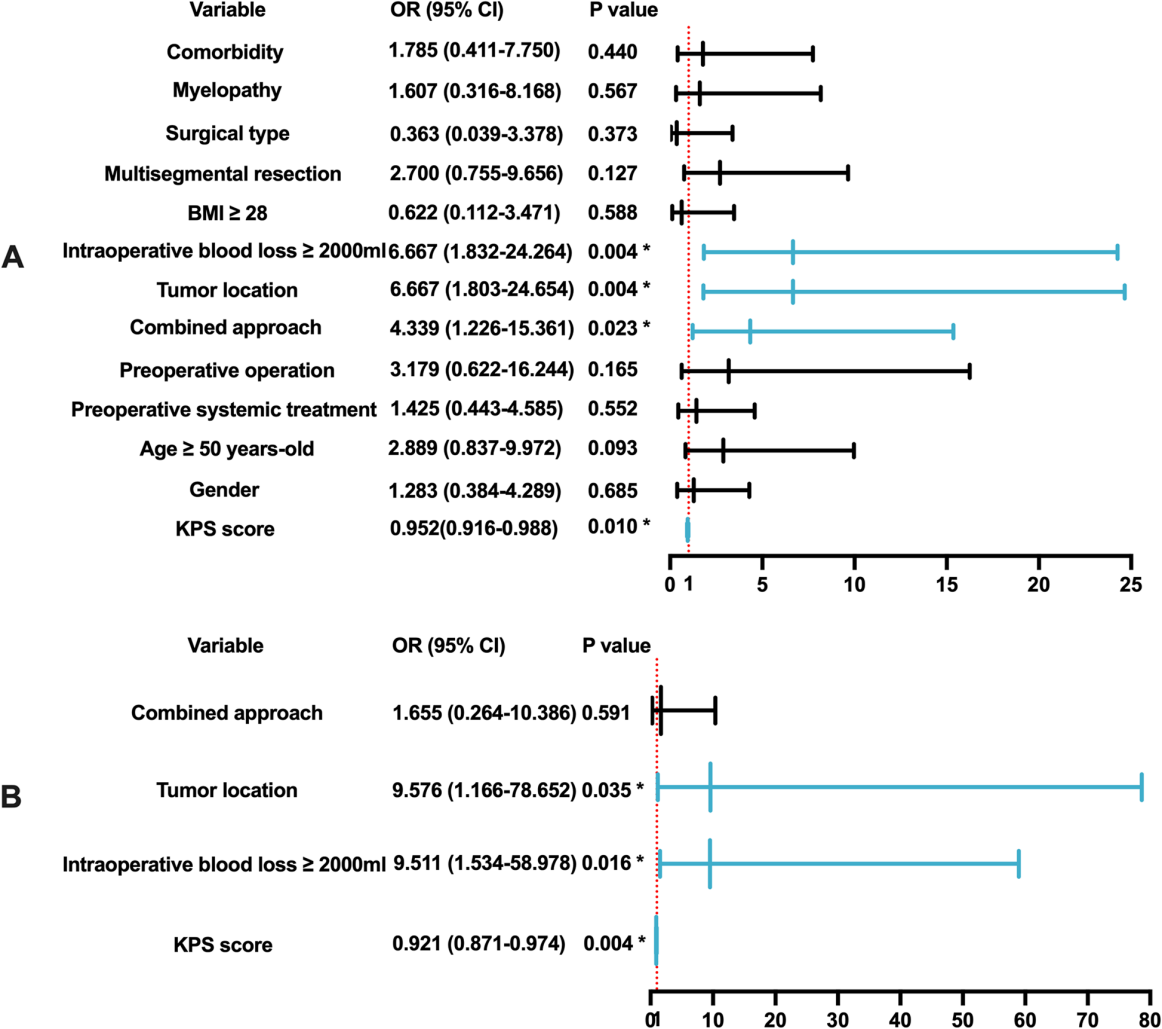
Predictors of major complications by univariate and multivariate analysis. **A** Forest plot of the results of the univariate analysis. **B** Forest plot of the results of the multivariate analysis. Abbreviations: OR, odds ratio; CI, confidence interval; BMI, body mass index; KPS, Karnofsky performance status

With regard to major complications, combined surgical approach (*P* = 0.023), low KPS score (*P* = 0.010), intraoperative blood loss ≥ 2000 mL (*P* = 0.004), and lumbar spine lesion (*P* = 0.004) were also associated with the occurrence of major complications, identified by the univariate analysis. In addition, major complications tended to be more frequent in patients aged > 50 years (*P* = 0.093); however, this difference was not statistically significant. Multivariate logistic regression analysis showed that independent predictors included a low KPS score (*P* = 0.004), lumbar spine lesion (*P* = 0.035), and massive intraoperative bleeding (*P* = 0.016). Major surgical complications were not significantly influenced by the surgical approach (Fig. 3).

The multivariate model used to predict wound complications included KPS score, multilevel resection, and intraoperative blood loss ≥ 2000 mL. A low KPS (*P* = 0.022) and massive intraoperative bleeding (*P* = 0.024) were independent predictors of wound complications. No significant relationship was found between multilevel resection (*P* = 0.321) and wound complications (Table 4).

**Table 4:** Predictors of wound complications by univariate and multivariate analysis

Factor	Univariate		Multivariate	
	OR (95% CI)	*P* value	OR (95% CI)	*P* value
Intraoperative blood loss (< 2000 ml vs. ≥ 2000 ml)	5.538 (1.257–24.396)	0.024	8.518 (1.325–54.745)	0.024
Multilevel resection (no vs. yes)	4.800 (1.163–19.805)	0.030	2.366 (0.432–12.949)	0.321
Baseline KPS	0.948 (0.911–0.987)	0.010	0.943 (0.897–0.992)	0.022
Age (< 50 years vs. ≥ 50 years)	1.333 (0.328–5.419)	0.688		
Gender (male vs female)	1.971 (0.454–8.551)	0.365		
Pre-op systemic treatment (no vs. yes)	2.229 (0.576–8.623)	0.246		
Pre-op operation (no vs. yes)	0.567 (0.061–5.277)	0.618		
Approach (postoperative vs. combined)	1.284 (0.291–5.656)	0.741		
Tumor location (thoracic vs. lumbar)	1.333 (0.328–5.419)	0.688		
BMI (< 28 kg/m^2^ vs. ≥ 28 kg/m^2^)	1.259 (0.216–7.326)	0.798		
Treatment type (SES vs. TES)	0.799 (0.073–6.702)	0.757		
Comorbidity (no vs. yes)	1.048 (0.185–5.943)	0.958		

*OR* odds ratio, *CI* confidence interval, *KPS* Karnofsky performance score, *BMI* body mass index, *SES* sagittal en bloc spondylectomy, *TES* total en bloc spondylectomy

**Fig. 4:**
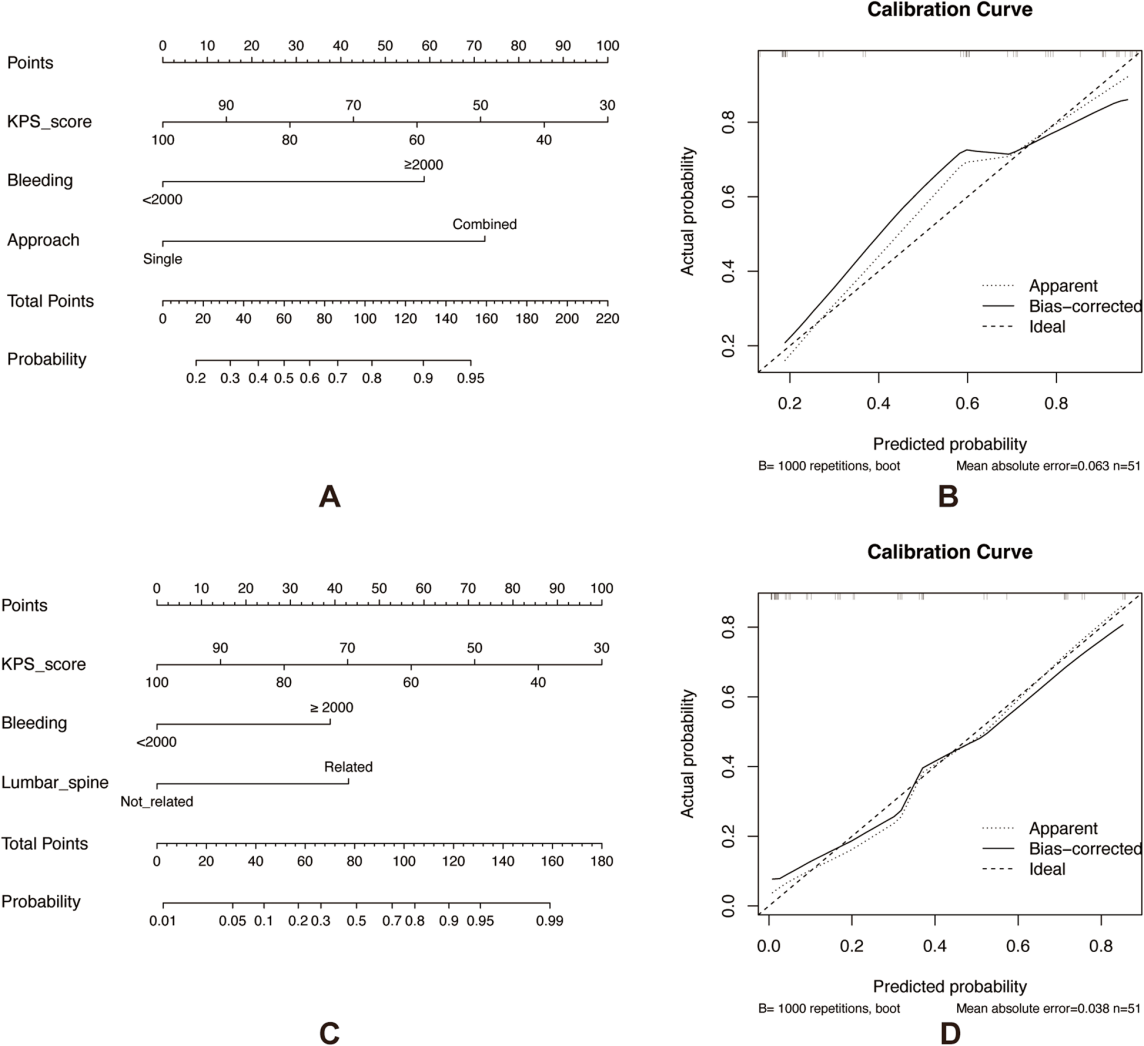
**A** Nomogram for prediction of overall complications after EBR. **B** Calibration plot of the nomogram for overall complications, the c-indices was 0.850. **C** Nomogram for prediction of major complications after EBR. **D** calibration plot of the nomogram for major complications, the c-indices was 0.891. Abbreviations: EBR, en bloc resection; KPS, karnofsky performance status

Furthermore, we constructed a nomogram to predict the overall and major complications using these significant variables. The internal validation results showed that the c-indices were 0.850 and 0.891 for overall and major complications, respectively, indicating good accuracy of the models (Fig. 4).

### Follow-up

**Fig. 5:**
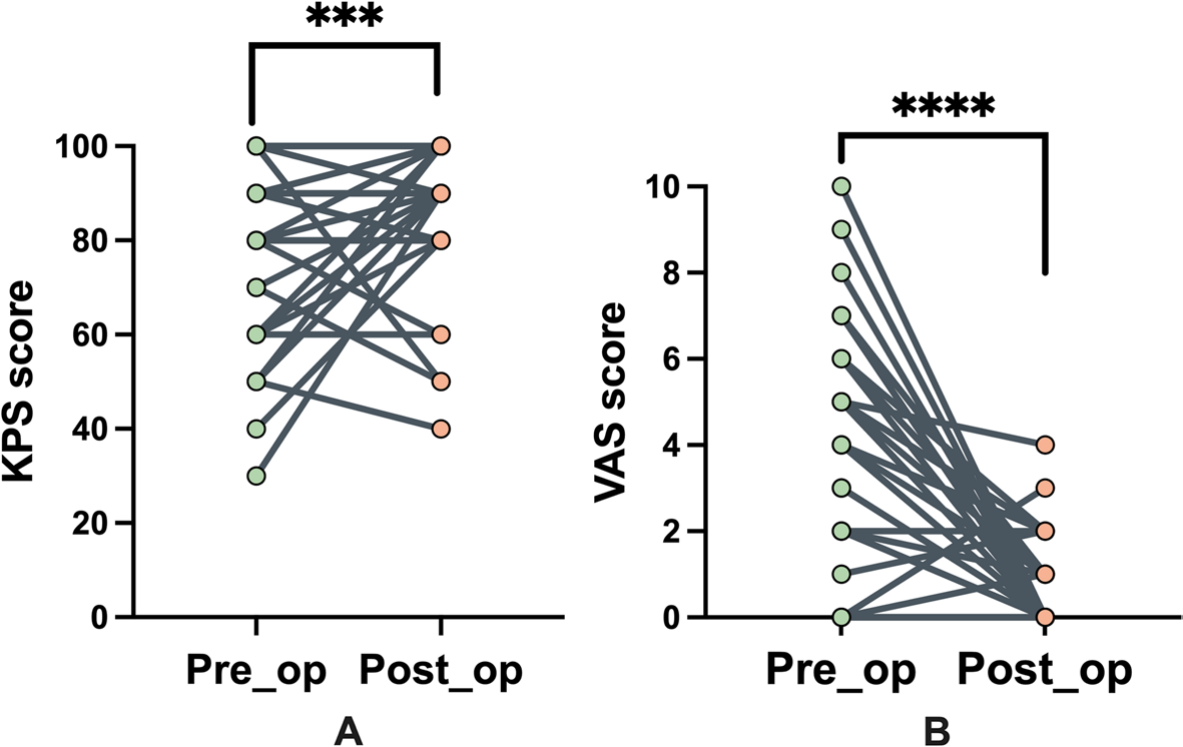
**A** Preoperative and postoperative KPS score of the patients. **B** Preoperative and postoperative VAS score of the patients. Abbreviations: Pre-op, preoperative; post-op, postoperative; KPS, karnofsky performance status; VAS, visual analog scale

**Fig. 6:**
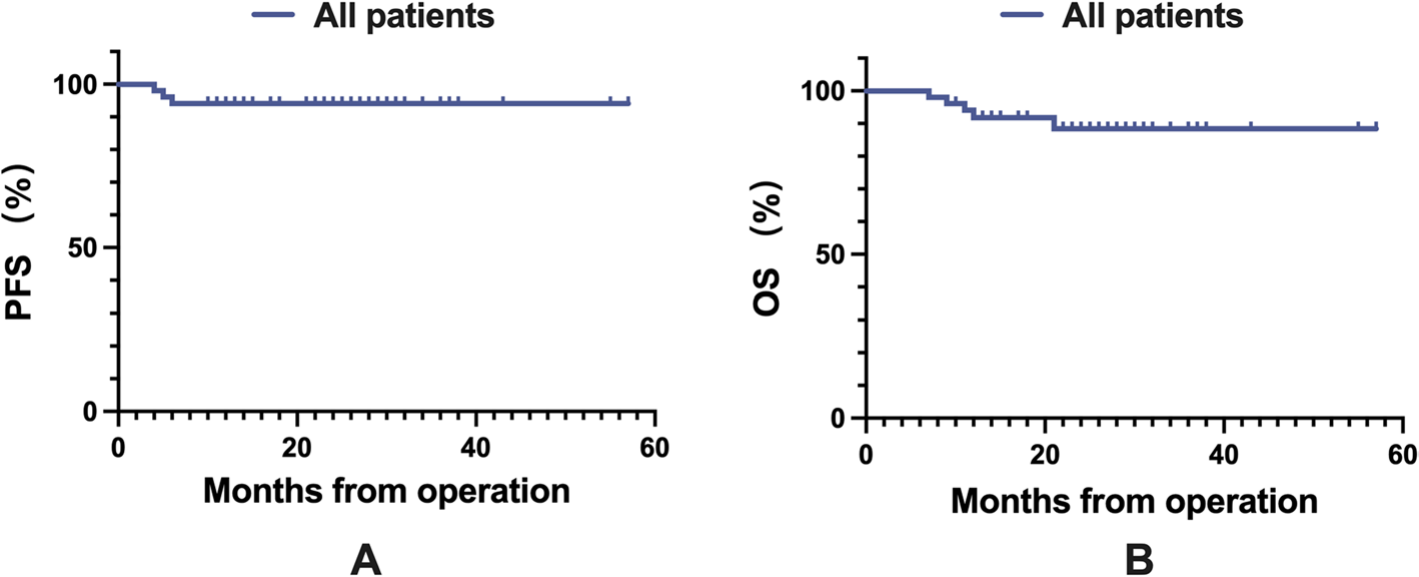
**A** Local control of the patients. **B** Overall survival of the patients. Abbreviations: PFS, progression-free survival; OS, overall survival

The median follow-up period was 21 (range, 7–57) months. VAS scores decreased significantly, and quality of life improved 3 months postoperatively (Fig. 5). Of the 7 patients with myelopathy, 5 experienced improvements in neurological function, while the other 2 remained almost unchanged. Three patients, 1 with osteosarcoma, 1 with chondrosarcoma, and 1 with myofibroblastic sarcoma, had radiographic evidence of tumor relapse throughout the follow-up period, all of whom were treated with intralesional resections, and the time to local recurrence was 5, 6, and 4 months, respectively. Systemic metastasis was detected in 16 patients. Five patients died at the time of the final follow-up, of whom 2 had evidence of local recurrence and the remaining 3 died from metastases without evidence of local failure at the time of death (Fig. 6).

## Discussion

EBR has been widely acknowledged as a standard treatment for malignant spinal tumors, with the advantage of complete tumor resection [1, 6, 13, 14]. Of note, the majority of previous reports discuss the use of traditional reconstruction techniques for anterior column reconstruction, of which the titanium mesh cage (TMC) is most commonly used. Durable spinal reconstruction is vital for the management of spinal tumors, and TMC can provide sufficient support and strength to effectively restore and maintain immediate spinal stability. Nevertheless, TMC has a sharp edge and cannot match the shape of the endplate and sagittal alignment of the spine, resulting in a reduced contact area, endplate fracture, implant collapse, and instrumentation failure, especially in patients with a long life expectancy [15, 16]. In a series of cases reported by Park et al. [17], the rod fracture rate after TES was approximately 37.5%. The advent of 3D-printed endoprostheses has simplified the operative procedure and revolutionized the reconstruction of spinal stability, which can facilitate bone growth owing to its precise shape matching and porous structure. Moreover, both the off-the-shelf and the customized 3D-printed AVB can increase the contact area with the adjacent endplate, reduce the pressure of the adjacent endplate, and enhance the stability of the internal fixation [15]. According to Zhou et al. [10], the overall fusion rate after reconstruction with a 3D-printed AVB was 87% at 37 months postoperatively, and only 2 out of 23 patients developed instrumentation-related complications. However, current research regarding 3D-printed AVB after EBR is mostly in the form of case reports or case series with a small sample size, and relevant surgical complications have rarely been reported.

To the best of our knowledge, this is the largest case series of 3D-printed AVB for anterior spine reconstruction reported to date. In our study, prosthesis mismatch occurred in only 2 patients reconstructed with the off-the-shelf 3D-printed AVB, and no pronounced subsidence was observed at the final follow-up. Customized 3D-printed AVB, which theoretically possessed high accuracy to precisely match the bone defect, exhibited excellent treatment outcomes to restore spinal alignment. Moreover, the overall instrumentation failure rate of our patients was 2.0% at a median follow-up of 21 months, much lower than that reported previously [2, 5], indicating that the 3D-printed AVB is an effective and reliable option for anterior reconstruction after EBR for thoracolumbar spinal tumors. Some surgeons worried about the manufacturing time of customized 3D-printed AVB could result in a delay to the surgical procedure [18]. However, this problem did not come up in our study and all patients underwent surgery without any delay.

In the present study, the overall complication rate was 56.9%, which is considerably high, and 16 of 51 patients experienced major complications. This is consistent with data from previous studies (Table 5), in which the overall complication rate ranged between 12.5% and 70.6% [5, 9, 13, 19–21]. The largest series examining TES for patients with spinal tumors included 307 patients, with 31.6% having multilevel lesions; perioperative complications were observed in 67.1% of patients, and multivariate analysis showed that the use of a combined surgical approach and multilevel TES was a significant independent factor [9]. Similar results were observed by Boriani et al. [22], who analyzed 220 patients treated with EBR, and 153 complications were detected in 100 patients (46.2%) following a median follow-up of 45 months. EBR is a beneficial but highly demanding procedure that often requires multidisciplinary collaboration and potentially results in an unacceptably high rate of complications [1, 2, 9, 23]. Therefore, it is important to identify the risk factors affecting complications to enhance perioperative management.

**Table 5:** Published reports of en bloc resection of the spine

Author/year	No. of patients/surgical type	Anterior reconstruction	Median F/ U (months)	Complication rate	Implant failure rate	LC rate
Disch 2011	20/EBS	Carbon composite cage	21.5	-	0%	95.0%
Matsumoto 2011	15/TES	TMC, ETC, cement	41.5*	-	40.0%	86.7%
Amendola 2014	103/EBR	Mesh, CF prosthetic system	-	41.7%	9.7%	78.6%
Boriani 2014	134/EBR	-	47.0	35.1%	-	84.3%
Wang 2016	17/EBR	TMC	24.0	70.6%	0%	70.6%
Shah 2017	33/TES	-	18.0	52.0%	25.0%	93.9%
Li 2019	30/TES	TMC	41.8*	-	26.7%	93.3%
Zoccali 2019	37/EBS	Cage, allograft shaft	42.2	64.9%	8.1%	82.4%
Park 2019	32/TES	TMC, ETC, bone graft	49.8*	-	37.5%	81.3%
Demura 2021	307/TES	-	-	67.1%	26.7%	89.6%
Hu 2022	8/TES	3D-printed AVB	11.5	12.5%	0%	100%
Present study 2023	51/EBR	3D-printed AVB	21.0	56.9%	2.0%	94.1%

*F/U* follow-up, *LC* local control, *EBS* en bloc spondylectomy, *TES* total en bloc spondylectomy, *TMC* titanium mesh cage, ETC expandable titanium cage, *EBR* en bloc resection, *CF* carbon fiber, *AVB* artificial vertebral body, *Mean follow-up

**Fig. 7:**
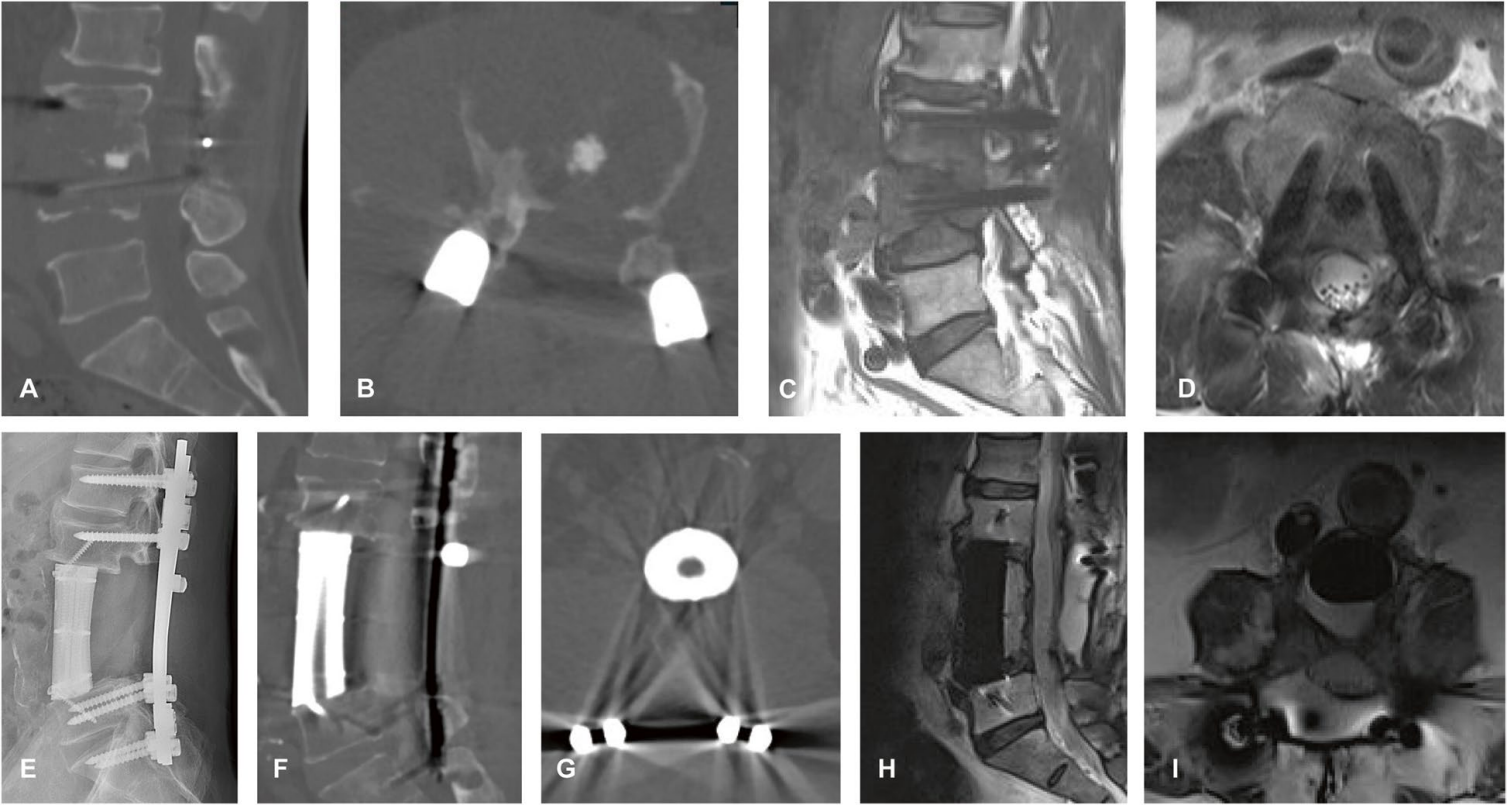
A 59-year-old man with a medical history of surgery for disc herniation complained of increasing back pain during the previous 1 month, his KPS score was 60, and the American Spinal Injury Association impairment scale was **D**. The patient underwent TES and spine reconstruction with customized 3D-printed AVB via a one-stage combined approach, and the estimated blood loss was 2400 mL. Intraoperatively, inferior vena cava injury was developed and repaired. **A**, **B** Preoperative CT scan revealed osteolytic body destruction in L3 and L4 vertebral. **C**,** D** Preoperative sagittal and axial T2-weighted MRI. **E** Postoperative lateral radiograph. **F**, **G** Postoperative CT scan showing an excellent position of the 3D-printed AVB. **H**,** I**, MRI scan at a 12-month follow-up showing no tumor recurrence. Abbreviations: KPS, Karnofsky performance status; TES, total en bloc spondylectomy; 3D, three-dimensional; AVB, artificial vertebral body; CT, computed tomography; MRI, magnetic resonance imaging

Several risk factors for overall and major complications were identified in our study, and the correlation between EBR of the lumbar spine and major complications is of particular interest. EBR in the lumbar spine is quite dangerous, and many difficulties, such as limited working spaces between the lumbar plexus and the tumor, may be encountered. Moreover, the paravertebral tumor component can become extremely large because of the large retroperitoneal space and invasion of critical organs and major vascular structures, potentially increasing operative difficulty [24]. Furthermore, EBR in the lumbar spine generally requires a combined surgical approach because of the vital and complex anatomy, which results in a longer operation time, massive blood loss, and higher complication rates (Fig. 7). In addition, we identified that a low KPS score and vast intraoperative bleeding were robust predictors of overall and major complications, consistent with previous reports. Yang et al. [25] retrospectively reviewed the course of 110 patients and found that a KPS score of < 60 and intraoperative blood loss of > 500 mL were significant risk factors for overall and major complications, respectively. However, these cases involved tumors in the cervical spine, and surgical strategies included both en bloc and intralesional resection.

**Fig. 8:**
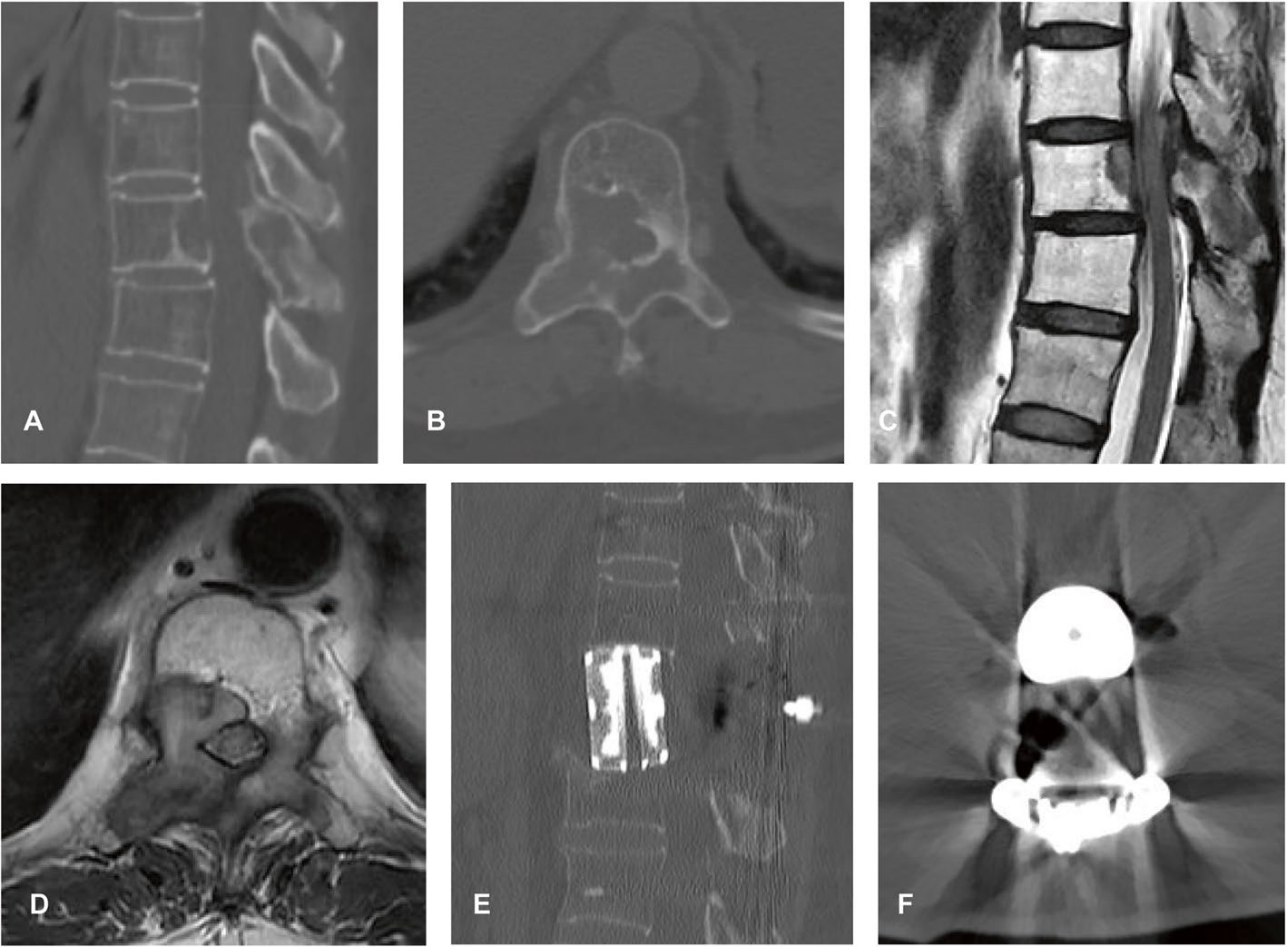
A 55-year-old woman with metastatic breast cancer experienced progressive weakness in the lower extremities for 3 months, her KPS score was 90, and the American Spinal Injury Association impairment scale was **E**. The patient underwent TES and spine reconstruction with off-the-shelf 3D-printed AVB via a posterior approach and the estimated blood loss was 1000 mL. The patient recovered well and no surgical complications occurred. **A**, **B** Preoperative CT scan showing the osteolytic body destruction of T10. **C**, **D** Sagittal and axial T2-weighted MRI showing epidural spinal cord compression. **E**, **F** Postoperative CT scan. Abbreviations: KPS, Karnofsky performance status; TES, total en bloc spondylectomy; 3D, three-dimensional; AVB, artificial vertebral body; CT, computed tomography; MRI, magnetic resonance imaging

Pulmonary complications, including pleural effusion and pneumonia, occurred the most frequently in our patients (*n* = 13, 25.4%). Similar to a previous study [9], surgery for thoracic spine tumors carried a higher risk for pulmonary complications than that for lumbar spine tumors (12.5% vs 31.4%). One possible reason for this difference is that thoracic spine surgery has a more direct surgical invasion, especially when the tumor is closely related to the lung. Wound-related complications were another common complication (*n* = 11, 21.5%) in this series and were also the major cause of revision surgery. EBR is a highly invasive procedure, often accompanied by a large wound and long operation time. Wound complications can contribute to longer hospital stays, unplanned reoperations, and even poor neurological outcomes [26]. Previous studies have shown correlations between prior irradiation and wound complications [27]. In our present study, however, prior irradiation was not a significant risk factor. Subgroup analysis identified low KPS score and intraoperative blood loss ≥ 2000 mL as significant risk factors for wound-related complications in the multivariate logistic regression analysis, which suggests that patients with good preoperative functional status and less invasive surgery will likely achieve better wound healing (Fig. 8).

The limitations of this study include its retrospective nature, inclusion of multiple tumor types, and single-institution analysis. Additionally, the follow-up duration was relatively short and late postoperative complications, including instrument failure, may take a long time to occur. Nevertheless, our study is one of the largest to investigate surgical complications in patients who underwent EBR with anterior spinal reconstruction using a 3D-printed AVB.

## Conclusions

In conclusion, a 3D-printed AVB is an effective and feasible reconstruction option for patients with thoracolumbar tumors treated with EBR. However, EBR has a steep learning curve and may be associated with a high complication rate. Combined surgical approach, low KPS score, and intraoperative blood loss ≥ 2000 mL were significant predictive factors for overall complications. The nomogram containing these factors provides an excellent model to predict complications. Thus, individualized surgical strategies should be established for high-risk patients to minimize complications.

## Data Availability

Data sharing is not applicable to this article as no datasets were generated or analyzed during the current study.

## Abbreviations

EBR: En bloc resection
3D: Three-dimensional
AVB: Artificial vertebral body
KPS: Karnofsky performance status
VAS: Visual analog scale
CT: Computed tomography
TES: Total en bloc spondylectomy
SES: Sagittal en bloc spondylectomy
LC: Local control
OS: Overall survival
TMC: Titanium mesh cage
